# MicroRNA‐224, negatively regulated by c‐jun, inhibits growth and epithelial‐to‐mesenchymal transition phenotype via targeting ADAM17 in oral squamous cell carcinoma

**DOI:** 10.1111/jcmm.14107

**Published:** 2019-06-17

**Authors:** Yaoyong Lu, Wendong Huang, Haiwen Chen, Huajun Wei, Aihua Luo, Guangsheng Xia, Xubin Deng, Gong Zhang

**Affiliations:** ^1^ Department of Oncology (Section 3) Gaozhou People’s Hospital Gaozhou China; ^2^ Department of Pharmacy Maoming People’s Hospital Maoming China; ^3^ Department of Pathology Gaozhou People’s Hospital Gaozhou China; ^4^ Department of Otolaryngology‐Head and Neck Surgery Gaozhou People’s Hospital Gaozhou China; ^5^ Affiliated Cancer Hospital & Institute of Guangzhou Medical University Guangzhou China; ^6^ Department of Radiotherapy People's Hospital of Shanxi Province Taiyuan China

**Keywords:** ADAM17, epithelial‐to‐mesenchymal transition, miR‐224, oral squamous cell carcinoma

## Abstract

Abnormal expression of miR‐224 has been reported to promote cancer progression. However, the role of miR‐224 is seldom reported in oral squamous cell carcinoma (OSCC). We reported that miR‐224 expression was significantly down‐regulated in OSCC tissues and cell lines. Restoration of miR‐224 decreased OSCC cell growth and invasion. In addition, luciferase and Western blot assays revealed that ADAM17 protein was a downstream target of miR‐224. The overexpression of ADAM17 dismissed miR‐224’s effect on cell growth and invasion. We concluded that miR‐224 inhibited OSCC cell growth and invasion through regulating ADAM17 expression. Subsequently, we revealed that c‐jun directly bind to miR‐224 promoter and decreased miR‐224 expression. Taken together, these findings demonstrated that miR‐224 may function as a tumour‐suppressive microRNA in OSCC and suggested that miR‐224 may be a potential therapeutic target for OSCC patients.

## 1 INTRODUCTION

Ranking the sixth most common cancer worldwide, oral squamous cell carcinoma (OSCC) accounts for >40% of head and neck malignancies.^1^ The OSCC often displays high rates of lymph node metastasis when diagnosed, which contributes to poor prognosis of the disease. Approximately up to 50% of OSCC patients were present with advanced disease and metastasis.^2^ Therefore, it is urgent to clarify the molecular pathogenesis underlying the aggressive progression of OSCC.

As a small and single‐stranded RNAs, microRNAs (miRNAs) bide at the 3'‐untranslated regions (UTRs) of target genes and negatively regulate the downstream genes expression. The role of miRNAs in regulating cancer progression has been fully investigated, including in OSCC.^3^, ^4^ The function of miR‐224 has been reported in a serious of cancer, including colorectal, breast, prostate, cervical and lung cancer.^5^, ^6^, ^7^, ^8^, ^9^, ^10^, ^11^ However, the underlying role of miR‐224 in human OSCC is still poorly understood.

ADAM17, as well as many other ADAM family members, is known to process single‐spanning membrane proteins such as cytokines, growth factors and regulators of cancer processes.^12^ Abnormal expression of ADAM17 is usually found in a series of cancer, such as ovarian cancer, colon cancer, breast cancer and OSCC.^13^, ^14^, ^15^ ADAM17 can be a novel therapeutic target for these cancers.

We revealed that miR‐224 was down‐regulated in OSCC tissues and cell lines. We also demonstrated that miR‐224 decreased growth and invasion in OSCC via targeting the ADAM17 protein. In particular, we uncovered that c‐jun decreased miR‐224 expression in OSCC by biding to its promoter. Targeting c‐jun/miR‐224/ADAM17 axis may be a promised strategy for OSCC.

## 2 MATERIALS AND METHODS

### 2.1 OSCC cell lines culture, cell transfection and OSCC patient samples

The two OSCC cell lines SCC‐9 and Cal‐27 were obtained from the Institute of Chemistry and Cell Biology of the Chinese Academy of Sciences (Shanghai, China). They were cultured in DMEM medium supplemented with 10% foetal bovine serum.

For transfection, cells were cultured to 80% confluence and transfected with miR‐224, miR‐224 inhibitor or miR‐ctrl using Lipofectamine 2000 (Invitrogen, Carlsbad, CA, USA) according to the manufacturer's recommendation.

The OSCC patient samples were collected from Gaozhou People's Hospital, Affiliated Cancer Hospital & Institute of Guangzhou Medical University and People's Hospital of Shanxi Province. All the experiments were performed in accordance with the approved guidelines of the Institutional Research Ethics Committee of these hospitals.

### 2.2 Real‐time PCR assay

Total RNA were extracted from OSCC cells and tissues by using TRIzol reagent (Invitrogen). Detection of miR‐224 levels was carried out by using the qRT‐PCR miRNA kit (Ruibo, Guangzhou, China).Under the ABI PRISM 7500 Sequence Detection System (ABI, Guangzhou, Guangdong Province, China), the real‐time PCR assay was performed by using a SYBR Green kit (TaKaRa, Tokyo, Japan). The primers used in the study were listed as follows: ADAM17 5'‐CACCAGAGACTCGAGAAGC‐3' (forward) and 5'‐TTTCTTACCGAATGCTGCTG‐3' (reverse); GAPDH 5'‐CCTCCTGTTCGACAGTCAG‐3' (forward) and 5'‐CATACGACTGCAAAGACCC‐3' (reverse); U6 5'‐ATACAGAGAAAGTTAGCACGG‐3' (forward) and 5'‐GGAATGCTTCAAAGAGTTGTG‐3' (reverse); miR‐224 5'‐GGGCAAGTCACTAGTGGTTC‐3' (forward) and 5'‐GTGCAGGGTCCGAGGT‐3' (reverse).

### 2.3 Production of lentivirus and cell infection

The plasmid carrying miR‐224 (cat. no. mh12808) and the control plasmid (cat. no. m001) were purchased from Applied Biological Materials (ABM, Richmond, Canada) Inc. We named this vector LV‐miR‐214 and the control plasmid was named LV‐ctrl. The package of viruses was carried out as the standard protocol. About 72 h later, the virus particles were harvested and stored in −80°C. Subsequently, cells were infected with virus particles and 8 μg/mL polybrene.

### 2.4 Colony formation and MTT assay

To perform colony formation assay, 200 cells were seeded in 6‐well culture plate and cultured for 2 weeks. After that, we washed the cells three times with PBS and stained them with Giemsa solution. Subsequently, the number of colonies containing ≥50 cells was counted under a microscope. The plate clone formation efficiency were evaluated by using the formula:  = (number of colonies/number of cells inoculated)×100%.

The MTT assay was carried out as previously described.^16^ Briefly, cells were seeded on 96‐well plate and were allowed to grow for 24 hours. The media was aspirated and MTT solution was added into each well. After incubated for 30 minutes, 150 µL DMSO was added into each well. Finally, the absorbance was read at OD = 590 nm.

### 2.5 Cell invasion ability assay

The cell invasion ability was examined by Boyden assay. The cells were seeded into the upper chambers (Millipore, Burlington, MA, USA) that coated with 150 µg Matrigel (BD Biosciences, Boston, MA, USA). Under the upper chambers were lower chambers that filled with 500 µL DMEM supplemented with 10% FBS. After incubation for 12 hours, the cells adhering to the lower surface were fixed with methanol, stained with Giemsa solution and counted.

### 2.6 Western blot assay

The total protein were extracted from cells with RIPA buffer (Beyotime, Beijing, China) and were then separated by SDS‐PAGE gel, followed by transferred to polyvinylidene fluoride (PVDF) membranes. We blocked the membranes by using 3% BSA/TBST and incubated them with primary antibodies at 4°C overnight. We then rinsed the PVDF membranes three times for 5 minutes with TBST and incubated them in HRP‐conjugated secondary antibodies for 1 hour at room temperature. We detected the levels of total protein were by using enhanced chemiluminescence reagents. The primary antibodies ADAM17 (lot. no. ab2051), C‐MYC (lot. no. ab39688), cyclind1 (lot. no. ab134175), CDK4 (lot. no. ab108357) and GAPDH (lot. no. ab9485) were purchased from abcam (Cambridge, UK). The concentration of the antibodies used in the study was 1:500. The primary antibodies E‐cadherin (lot. no. sc‐71009), N‐cadherin (lot. no. sc‐53488) and Vimentin (lot. no. sc‐73258) were purchased from Santa Cruz (Santa Cruz, CA, USA). The concentration of the antibodies used in the study was 1:500.

### 2.7 Luciferase reporter assay

We cloned the full‐length ADAM17 cDNA (lacking the 3′‐UTR) into the eukaryotic expression vector pcDNA3.1 (Invitrogen). Subsequently, the 3'‐UTR untranslated region of ADAM17 was amplified and cloned downstream of the firefly luciferase gene in the pGL3 vector (Promega, Madison, WI, USA) and the vector as named wild‐type (WT) ADAM17‐3'‐UTR. By using GeneTailor Site‐Directed Mutagenesis System (Invitrogen), we made site‐directed mutagenesis of the miR‐224 biding sites in the ADAM17 3'‐UTR. The vector was named mutant type (MUT) ADAM17‐3'‐UTR. Subsequently, we cotransfected the OSCC cells with the wt or mut ADAM17‐3′UTR vector and miR‐224 mimic or inhibitor. Finally, we performed the luciferase assay by using the dual Luciferase reporter assay system (Promega) 36 hours after transfection. To perform miR‐224 promoter luciferase assays, OSCC cells were seeded into 24‐well plates and co‐transfected with plasmids that contains miR‐224 promoter and the pRL‐TK‐Renilla plasmid (Promega).

### 2.8 Chromatin immunoprecipitation** assay**

We performed the chromatin immunoprecipitation (ChIP) assay according to the manufacturer's instructions by a ChIP assay kit (Millipore, catalog: 17‐371). Briefly, the cells were fixed with 1% formaldehyde to covalently crosslink proteins to DNA followed by harvesting chromatin from the cells. Subsequently, the crosslinked DNA (sheared to 200‐1000 base pairs in length) that linked with sonication were processed to an immmmunoselection process. Then the PCR assay was performed to measure enrichment of DNA fragments in the putative c‐JUN‐binding sites in the miR‐224 promoter.

### 2.9 Tumour xenograft experiments and Ki‐67 staining assay

The female BALB/c nude mice (5‐week‐old) were fed under standard conditions and cared according to the institutional guidelines for animal care. The animal experiments were approved by the Institutional Animal Care and Use Committee of Gaozhou People's Hospital. The LV‐miR‐224 and LV‐miR‐ctrl cells were injected subcutaneously into the posterior flank of the mice. Five mice were used in each group. We calculated tumour volumes by using the formula (volume = length × width2/2). Five weeks after the implantation, the xenografts were removed from the mice and the xenografts were weighed. Then the Ki‐67 stain assay was carried out to evaluate the proliferation index. Briefly, the paraffin‐embedded tissue sections were incubated with Ki‐67 antibody (1:200 dilutions) overnight at 4˚C, followed by a biotin‐labelled secondary antibody (1:100 dilutions) for 1 h at room temperature. Sections were incubated with ABC‐peroxidase and diaminobenzidine, counterstained with haematoxylin and visualized using light microscopy.

## 3 RESULTS

### 3.1 The expression of miR‐224 in OSCC tissues

**Figure 1:**
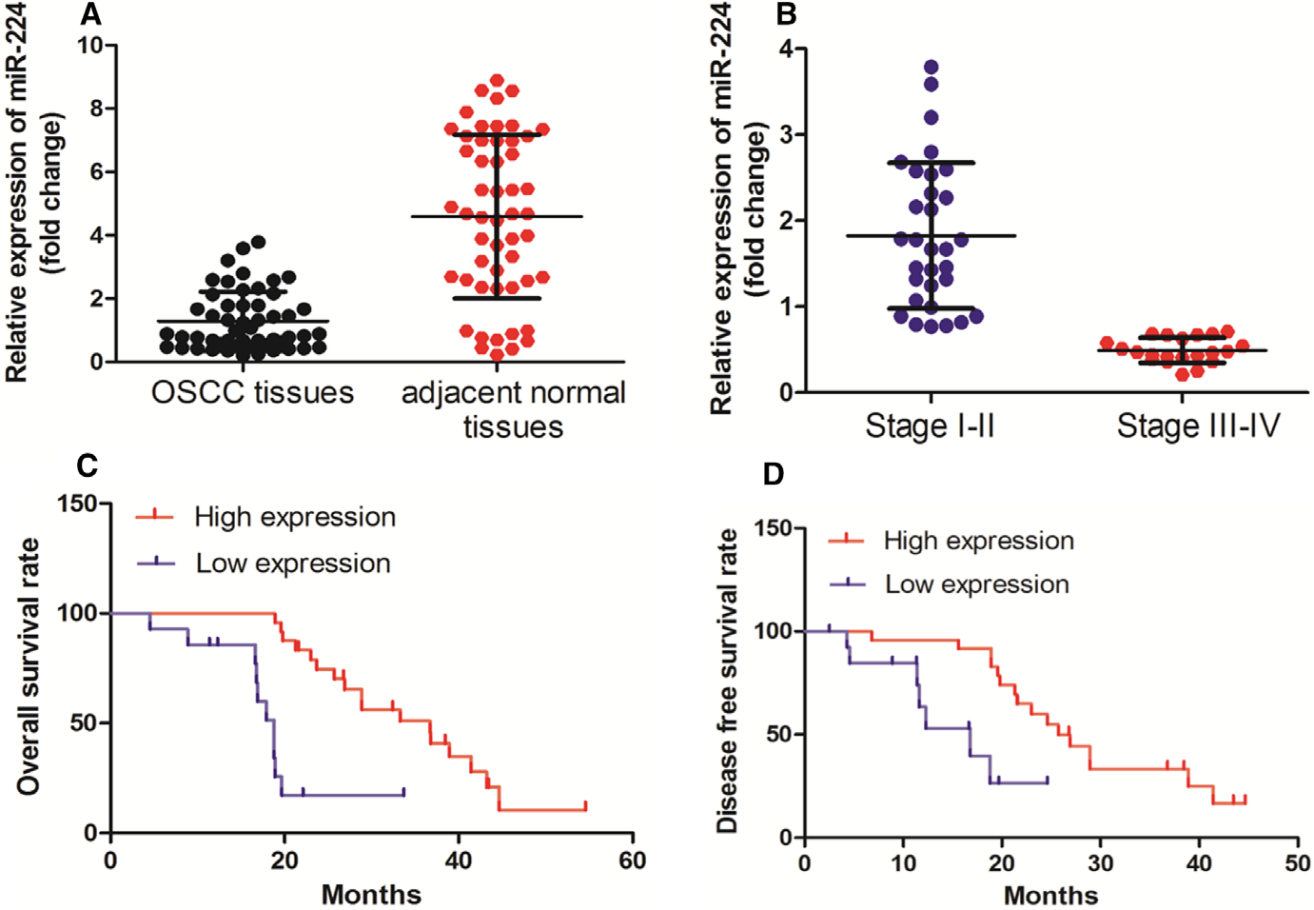
miR‐224 expression was down‐regulated in OSCC tissues. A, miR‐224 expression was significantly decreased in OSCC tissues vs corresponding non‐tumour tissues, as determined by RT‐PCR. B, miR‐224 expression was negatively associated with OSCC clinical stage. C, OSCC patients with lower levels of miR‐224 had shorter overall survival when compared those with higher levels of miR‐224. D, Patients with higher miR‐224 expression had better disease free survival rate than those with lower miR‐224 expression

We performed RT‐PCR assay to examine the level of miR‐224 expression in OSCC tissues. It was found that the expression of miR‐224 was significantly down‐regulated in OSCC tissues when compared with that in adjacent normal tissues (Figure 1A, *P* < 0.05). Interestingly, we revealed that the miR‐224 expression was negatively associated with OSCC clinical stage (Figure 1B, *P* < 0.05).

### 3.2 Correlation of miR‐224 expression with clinicopathological factors in OSCC

Subsequently, the correlation of miR‐224 expression with clinicopathological factors in OSCC patients was evaluated. There was no significant difference between miR‐224 expression level and gender, age and histological grade. However, we revealed that miR‐224 down‐regulation was associated with T stage (*P* = 0.000), Lymphatic invasion metastasis = (*P* = 0.006) and Distant metastasis (*P* = 0.009) (Table 1). Next, we explored the relation between OSCC patients overall survival rate, disease free survival rate and miR‐224 expression. It was found that OSCC patients with lower levels of miR‐224 had shorter overall survival and disease free survival rate than those with higher levels (Figure 1C,D).

**Table 1:** Correlation between miR‐224 expression and clinicopathological profiles

MiR‐224 expression		Clinicopathological profiles	n	*P* value
High	Low	*Gender*		
20	17	Male	36	0.463
24	28	Female	52	
		*Age*		
25	21	<60	45	0.338
19	24	≥60	43	
		*Histological grade*		
21	25	Moderate+poor	46	0.460
23	20	well	42	
		*T stage*		
34	16	T1‐T2	49	0.000
10	29	T3‐T4	39	
		*Lymphatic invasion*		
29	17	Negative	45	0.006
15	28	Positive	43	
		*Distant metastasis*		
12	24	Yes	36	0.009
32	21	No	52	

### 3.3 ADAM17 was identified as a downstream target of miR‐224

**Figure 2:**
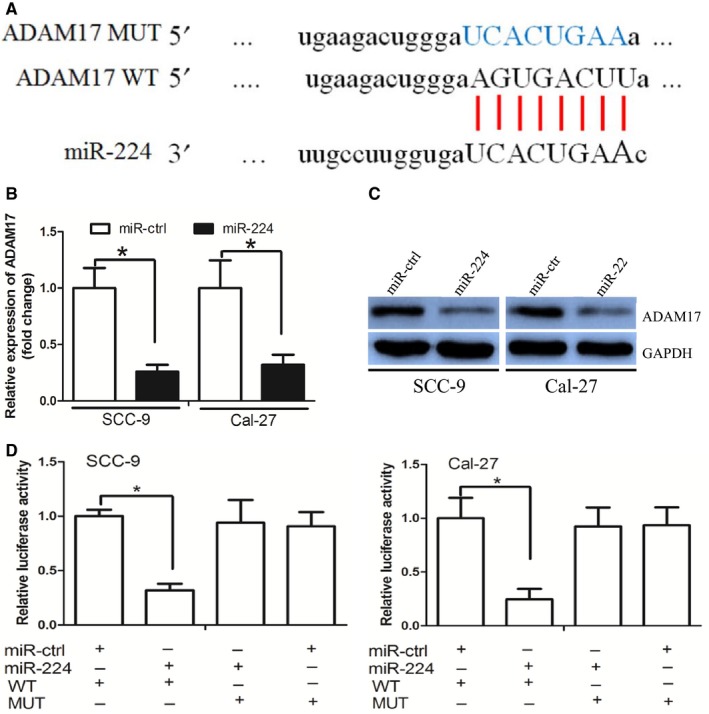
miR‐224 directly targeted ADAM17. A, ADAM17 wild‐type (WT) and mutant (MUT) 3′‐UTR as indicated. B and C, miR‐224 decreased ADAM17 expression at mRNA and protein level, respectively. D, miR‐224 decreased the luciferase activity of ADAM17 WT 3′‐UTR instead of MUT 3′‐UTR in OSCC cells

MicroRNAs exert its function through targeting their targets and we searched the potential targets of miR‐224 by TargetScan and miRanda. The ADAM17 protein was identified as a potential target of miR‐224 (Figure 2A). ADAM17 functions as an oncogene and can be a potential therapeutic target in oral cancer, we thus chose it for further study.^17^, ^18^ The RT‐PCR and Western blot assay demonstrated that miR‐224 inhibited ADAM17 mRNA and protein expression, respectively (Figure 2B,C). We performed luciferase reporter assay to determine whether miR‐224 directly targeted 3′‐UTR region of ADAM17. The 3′‐UTR region of ADAM17 mRNA including the predicted miR‐224 recognition site (WT) or the mutated sequence (mutant type) were subcloned into luciferase reporter plasmids (Figure 2A). We revealed that miR‐224 decreased luciferase activity in the WT vector, but not that in the mutant type vector (Figure 2D).

### 3.4 ADAM17 mediated miR‐224’s effect on cell growth and invasion

**Figure 3:**
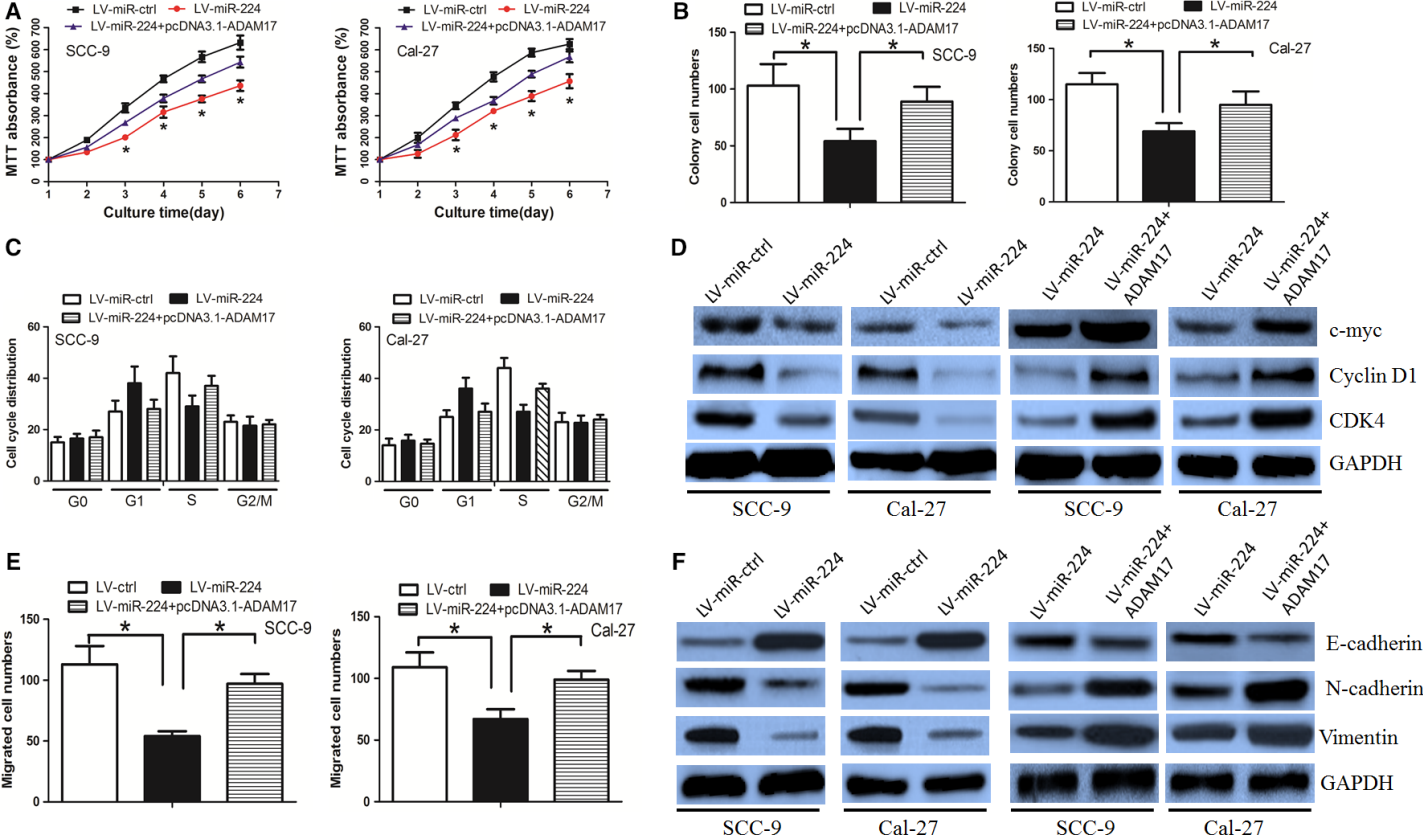
ADAM17 mediated miR‐224’s effect on cell growth and invasion. A, MiR‐224 decreased OSCC cell growth, while overexpression of ADAM17 counteracted this effect, as determined by MTT assay. B, MiR‐224 impaired OSCC cell colony formation ability, while ADAM17 restoration counteracted the effect. C, MiR‐224 delayed cell cycle progression from the G1 phase to S phase whereas this effect was dismissed by ADAM17 restoration. D, MiR‐224 negatively regulated G1/S phase checkpoint proteins and ADAM17 overexpression counteracted these effects. E, MiR‐224 decreased cell invasion ability, which was offset by ADAM17 overexpression. F, MiR‐224 inhibited the EMT phenotype, while the effect was neutralized by ADAM17 overexpression

First, we established OSCC cells stably expressing miR‐224 by using lentiviral vector‐mediated overexpression (LV‐miR‐224). Cells were also transduced with a control lentiviral vector (LV‐ctrl). The efficiency of transduction was validated using RT‐PCR (Figure S1A). The cell viability was decreased in LV‐miR‐224 group when compared with that in LV‐ctrl group, as determined by the MTT assay (Figure 3A). In parallel, the LV‐miR‐224 cells formed smaller and fewer colonies than the LV‐ctrl cells (Figure 3B). The EdU labelling assay further confirmed that miR‐224 decreased OSCC cell proliferation ability (Figure S1B). We then asked whether miR‐224 affected cell growth via altering cell cycle progression. We observed a lower proportion of S phase and a higher proportion in G1 phase in LV‐miR‐224 cells compared with that in LV‐ctrl cells (Figure 3C). In addition, the expression of G1/S phase checkpoint proteins such as cyclin D1, CDK4 and c‐myc were significantly decreased in LV‐miR‐224 cells, as determined by Western blotting assay (Figure 3D). Our findings demonstrated that miR‐224 inhibited OSCC cell growth by affecting cell cycle progression from the G1 phase to S phase.

Subsequently, we asked whether miR‐224 regulated cell invasion ability. We revealed that miR‐224 decreased cell invasion ability, as determined by the boyden assay (Figure 3E, Figure S1C). We further explored whether miR‐224 inhibited the EMT phenotype, which was responsible for cancer cell invasion. It was observed that the expression of the epithelial marker E‐cadherin increased, whereas expression of the mesenchymal markers N‐cadherin and Vimentin decreased in LV‐miR‐224 cells, as determined by the Western blot assay (Figure 3F). In all, these data demonstrated that miR‐224 inhibited EMT phenotype in the OSCC cells.

We also performed rescue experiment to determine whether miR‐224 exerted its function mainly through ADAM17. It was revealed that the overexpression of ADAM17 counteracted miR‐224’s effect on cell growth, cell cycle distribution, invasion and EMT phenotype (Figure 3A‐F).

Taken together, our findings revealed that miR‐224 decreased OSCC cell growth and invasion ability through regulating ADAM17 expression.

### 3.5 c‐jun decreased miR‐224 expression through binding at its promoter

**Figure 4:**
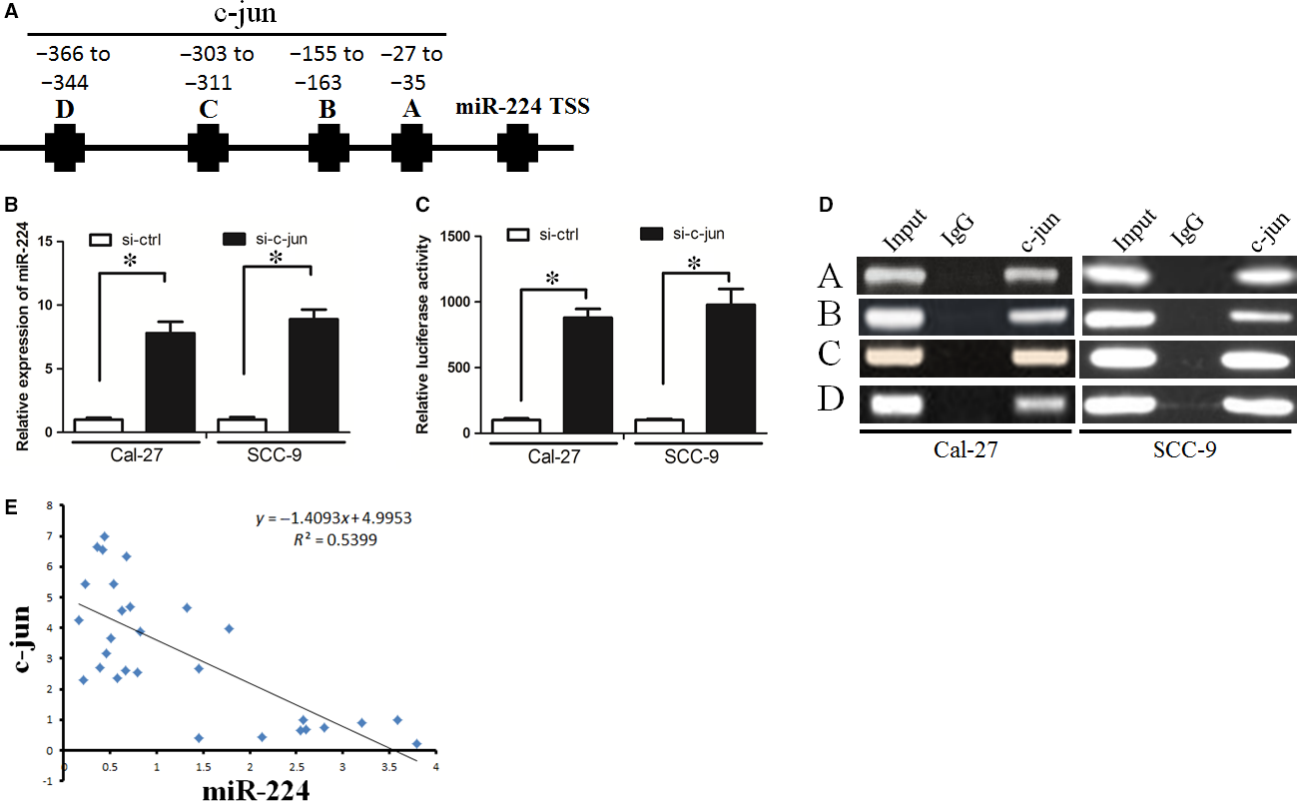
c‐jun decreased miR‐224 expression through biding at its promoter. A, The putative transcription factor‐binding sites of c‐jun at miR‐224 promoter region. B, c‐jun down‐regulation increased miR‐224 expression. C, c‐jun down‐regulation increased miR‐224 promoter luciferase activity. D, The CHIP assay confirmed that c‐jun protein was recruited to all the four binding sites in the putative miR‐224 promoter. E, miR‐224 expression was negatively correlated with c‐jun expression in OSCC tissues

We used UCSC and PROMO bioinformatics software to analyse a 0.8‐kb region upstream of the transcription start site of miR‐224. Four c‐jun‐binding motifs at −27 to −35, −155 to −163, −303 to −311 and −366 to −344 were identified inside the putative promoter region upstream of the miR‐224 transcriptional start site. We named these transcription factor‐binding sites (TFBSs) A, B, C and D (Figure 4A). Subsequently, we used si‐RNAs to knock‐down c‐jun expression in OSCC cells and found that miR‐224 expression was significantly increased in these cells when c‐jun was down‐regulated (Figure 4B). In addition, c‐jun down‐regulation increased miR‐224 promoter luciferase activity (Figure 4C). Finally, the ChIP assay confirmed that c‐jun protein was recruited to all the four binding sites in the putative miR‐224 promoter in Cal‐27 and SCC‐9 cells (Figure 4D). We further revealed that miR‐224 expression was negatively correlated with c‐jun expression (Figure 4E, Spearman's correlation coefficient, *R* = −0.7635).

### 3.6 Restoration of miR‐224 decreased cell growth in vivo

**Figure 5:**
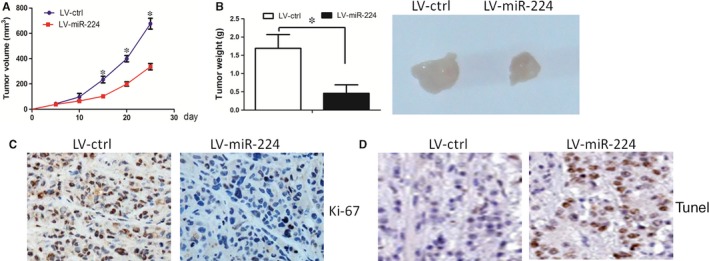
Restoration of miR‐224 decreased cell growth in vivo. A, LV‐miR‐224 cell‐derived xenograft tumours grew more slowly when compared with the LV‐ctrl cell‐derived xenograft tumours. B, The mean weight of LV‐miR‐224 cell‐derived xenograft tumours was less when compared with LV‐ctrl cell‐derived xenograft tumours. C, The Ki‐67 staining assay also revealed that LV‐miR‐224 cells had less proliferation index than the LV‐ctrl cells. D, The tunel assay revealed that the apoptosis rate was increased in LV‐miR‐224 cells, when compared with that in LV‐ctrl cells

We further asked whether miR‐224 increased cell growth in vivo. LV‐miR‐224 or LV‐ctrl cells were inoculated into the back of the nude mice, respectively. Compared with LV‐ctrl cell‐derived xenograft tumours, LV‐miR‐224 cell‐derived xenograft tumours grew more slowly when compared with the LV‐ctrl cell‐derived xenograft tumours (Figure 5A). In addition, the mean weight of LV‐miR‐224 cell‐derived xenograft tumours was less when compared with LV‐ctrl cell‐derived xenograft tumours (Figure 5B). Interestingly, the Ki‐67 staining assay also revealed that LV‐miR‐224 cells had less proliferation index than the LV‐ctrl cells (Figure 5C). Taken together, these results suggested that miR‐224 decreased OSCC cells growth in vivo.

## 4 DISCUSSION

OSCC often develop cervical lymph node and distant organ metastasis due to its aggressive characteristics. Thus, it is urgent to elucidate the molecular mechanisms underlying OSCC tumorigenesis. MiRNAs have played significant roles in the invasion and metastases of malignant tumour cells. Currently, a list of oncogenic miRNAs is considered to be potential serological markers of OSCC.^19^ It has been reported that miR‐224 may function as a tumour‐suppressive miRNA or an oncogenic miRNA depending on the tumour type. In colorectal cancer, miR‐224 can sustain Wnt/β‐catenin signalling and subsequently improve cell aggressive phenotype.^20^ Similarly, other researchers have also observed that miR‐224 promoted colorectal cancer cell proliferation and invasion. In addition, miR‐224 could be a suitable maker that predicted relapse of colorectal cancer.^10^ In parallel, miR‐224 was elevated in hepatocellular carcinoma and its overexpression contributed to G1/S checkpoint release and led to accelerating cell growth.^21^ MiR‐224 was increased in advanced melanoma expression and drove EMT through TXNIP down‐regulation.^22^ In addition, up‐regulation of miR‐224 was associated with aggressive progression and poor prognosis in human cervical cancer.^23^ However, miR‐224 functioned as a tumour‐suppressor in some types of cancer. For instance, miR‐224 decreased cell proliferation and migration via down‐regulating Fizzled 5 expression in breast cancer.^24^ Restoration of miR‐224 inhibited cell proliferation, migration and invasion in osteosarcoma.^25^ MiR‐224 inhibited cancer cell migration and invasion via targeting oncogenic TPD52 in prostate cancer.^26^ However, the role of miR‐224 in OSCC has seldom been investigated.

In this study, we reported that miR‐224 expression was down‐regulated in OSCC tissues. Subsequently, we uncovered that miR‐224 was associated with T stage, Lymphatic invasion metastasis and distant metastasis. Interestingly, the OSCC patients with higher levels of miR‐224 had higher overall survival and disease free survival rate than those with lower levels. Our data suggested that miR‐224 may function as a tumour suppressor in OSCC.

In the functional experiment, we revealed that miR‐224 overexpression decreased OSCC cell growth and invasion. The MTT and colony formation assays demonstrated that miR‐224 decreased cell growth ability in OSCC. The cell‐cycle progression is a predominant factor promoting tumour cell growth. We observed that miR‐224 delayed cell cycle from G1 phase to S phase and proposed that miR‐224 may decrease OSCC cell growth ability through affecting cell cycle distribution. In parallel, the in vivo study revealed that miR‐224 inhibited cell growth in the nude mice, which consolidated the data from the in vitro study.

Subsequently, we revealed that miR‐224 decreased OSCC cell invasion ability by using boyden assay. The EMT phenotype is tightly associated with cell invasion ability and we thus asked whether miR‐224 inhibited EMT phenotype. The Western blot and immunofluorescence assays demonstrated that miR‐224 increased E‐cadherin expression while decreased N‐cadherin and Vimentin expression. We speculated that miR‐224 may inhibit EMT phenotype and ultimately decreased cell invasion.

Emerging evidence demonstrated the role of ADAM17 in cancer progression, invasion and metastasis.^27^ ADAM17 promoted cell proliferation through accelerating cell cycle from G1 to S phase by increasing CDK2, cyclin E and decreasing p21 and p27 proteins.^28^ In gastric carcinoma, ADAM17 promoted epithelial‐mesenchymal transition via TGF‐β/Smad pathway.^29^ These data suggested that ADAM17 may promote cancer progression through affecting cell growth and invasion. A previous study confirmed that overexpression of ADAM17 contributed to OSCC development and targeting ADAM17 may be a potential therapeutic target in OSCC.^17^ Our findings revealed that miR‐224 inhibited OSCC cell growth and invasion, whereas, restoration of ADAM17 counteracted miR‐224’s effect. These findings suggested that ADAM17 may mediate miR‐224’s function in OSCC.

Subsequently, we explored the underlying mechanism that contributed to the dysregulation of miR‐224 in OSCC. Recruitment of specific transcription factors often led to abnormal miRNAs expression at genetic or epigenetic levels.^30^ The transcription factor c‐jun is a key regulator of cell growth 31 and metastasis 32 in cancer. We revealed four putative biding sites of c‐jun in the region upstream of miR‐224 locus. The subsequent experiment demonstrated that c‐jun could negatively regulate miR‐224 expression by directly biding at its promoter. We further confirmed that there was a negative correlation between c‐jun and miR‐224 in the OSCC tissues. Taken together, our findings revealed that c‐jun was responsible for miR‐224 down‐regulation in OSCC. Alternatively, there may be some other factor that may contribute to down‐regulation of miR‐224. For instance, the promoter methylation usually led to the abnormal expression of miRNAs.^33^ Whether promoter methylation is also responsible for miR‐224 down‐regulation needs further study.

In all, our data provide the first evidence that the c‐jun/miR‐224/ADAM17 axis controls cell growth and invasion in OSCC cells. Since down‐regulation of miR‐224 associates with poor prognoses and restoration of miR‐224 decreased cell growth and invasion ability, therapeutics that targeting miR‐224 may improve the treatment of OSCC.

## CONFLICT OF INTEREST

None declared.
