# Supplementary figures and images for: MicroRNA‐224, negatively regulated by c‐jun, inhibits growth and epithelial‐to‐mesenchymal transition phenotype via targeting ADAM17 in oral squamous cell carcinoma
Source: J Cell Mol Med. 2019 Jun 17;23(8):4913–20. doi: 10.1111/jcmm.14107 (PMC6653679; doi:10.1111/jcmm.14107)

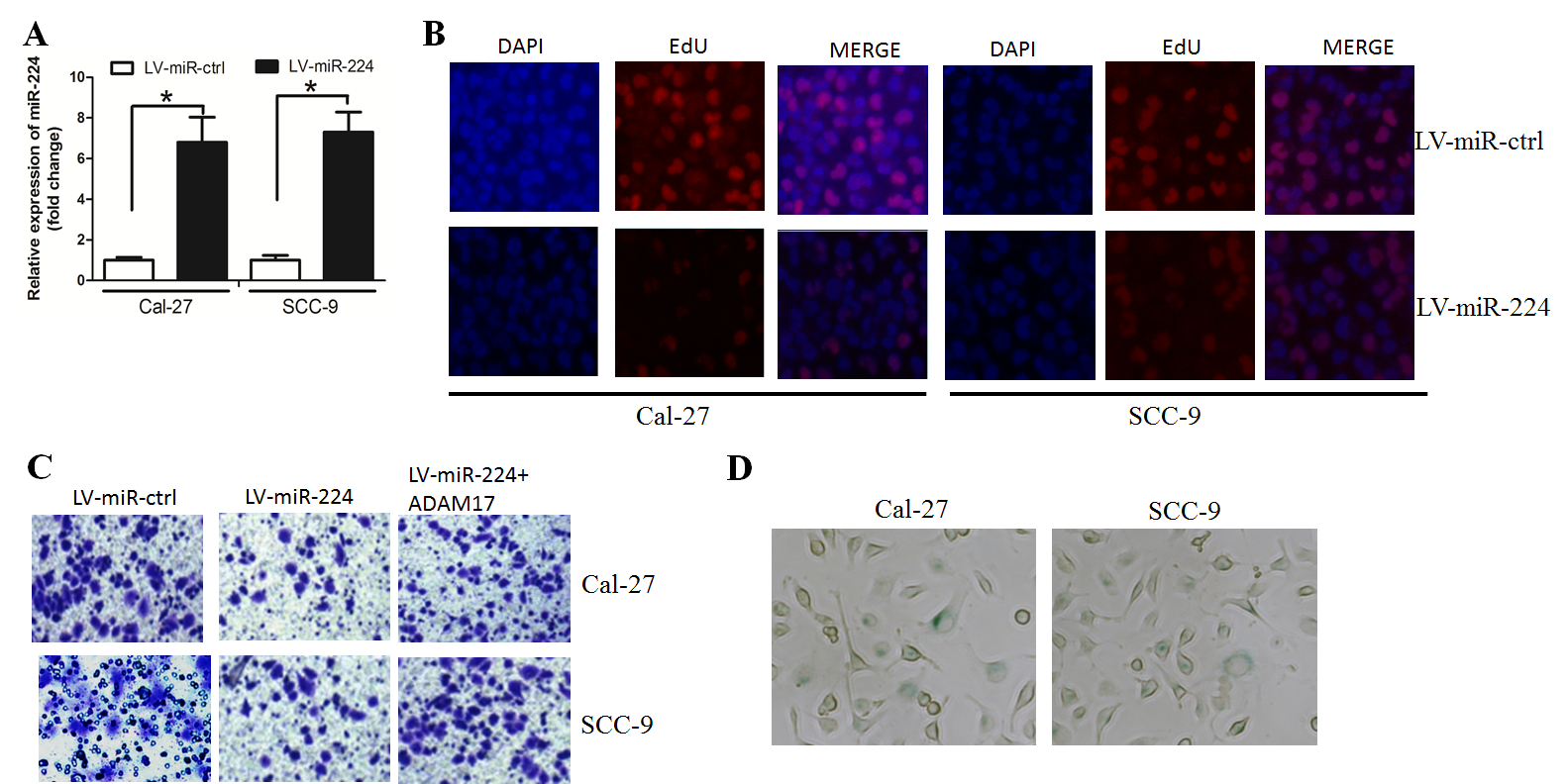

Supplement: Supplementary file 1 [file JCMM-23-4913-s001.tif]
